# Introducing an osteopathic approach into neonatology ward: the NE-O model

**DOI:** 10.1186/2045-709X-22-18

**Published:** 2014-05-09

**Authors:** Francesco Cerritelli, Marta Martelli, Cinzia Renzetti, Gianfranco Pizzolorusso, Vincenzo Cozzolino, Gina Barlafante

**Affiliations:** 1 EBOM – European Institute for Evidence Based Osteopathic Medicine, Pescara, Italy; 2 AIOT – Accademia Italiana Osteopatia Tradizionale, Pescara, Italy

**Keywords:** Complementary and alternative medicine, Integrated medicine, Neonatology intensive care unit, Newborns, Osteopathic manipulative treatment

## Abstract

**Background:**

Several studies showed the effect of osteopathic manipulative treatment on neonatal care in reducing length of stay in hospital, gastrointestinal problems, clubfoot complications and improving cranial asymmetry of infants affected by plagiocephaly. Despite several results obtained, there is still a lack of standardized osteopathic evaluation and treatment procedures for newborns recovered in neonatal intensive care unit (NICU). The aim of this paper is to suggest a protocol on osteopathic approach (NE-O model) in treating hospitalized newborns.

**Methods:**

The NE-O model is composed by specific evaluation tests and treatments to tailor osteopathic method according to preterm and term infants’ needs, NICU environment, medical and paramedical assistance. This model was developed to maximize the effectiveness and the clinical use of osteopathy into NICU.

**Results:**

The NE-O model was adopted in 2006 to evaluate the efficacy of OMT in neonatology. Results from research showed the effectiveness of this osteopathic model in reducing preterms’ length of stay and hospital costs. Additionally the present model was demonstrated to be safe.

**Conclusion:**

The present paper defines the key steps for a rigorous and effective osteopathic approach into NICU setting, providing a scientific and methodological example of integrated medicine and complex intervention.

## Background

Osteopathy is a manual medicine classified as complementary and alternative medicine (CAM). It is based on manual contact for diagnosis and treatment. It stresses the importance on the structural and functional integrity of the body as well as the intrinsic ability of the body for self-healing. Osteopaths can use numerous manual techniques to treat somatic dysfunction (SD) (ICD-10-CM Diagnosis Code M99.0-9) in order to enhance physiological functions [1]. Some studies and case reports starting shedding positive light on the effect of OMT on neonatal care. Lund et al. [2] and Wescott [3] showed a possible effect of osteopathic procedures in the management of sucking dysfunctions. Similarly, Andreoli et al. [4] suggested that OMT can be used as an adjuvant treatment for clubfoot complications, whilst Friedman [5] corroborated the application of osteopathy in reducing gastrointestinal problems, such as colics and regurgitation. Moreover, OMT seems to have a role in improving cranial asymmetry of infants affected by plagiocephaly [6]. Two studies demonstrated the efficacy of OMT in reducing length of stay and gastrointestinal function in newborns [7,8]. Authors claimed that the use of OMT is clinically effective in improving infant health status.

The osteopathic scenario, however, lacks standardized guidelines for the evaluation and treatment of newborns. Even more uncertainty is present if osteopathic care within a NICU environment is considered.

The aim of this paper is to describe a specific osteopathic approach to evaluate and treat hospitalized neonates. Since the attention to the care of neonates osteopathically treated is increased, we considered the need to address the lack of a standardized approach, based upon our experience of osteopathy in neonatology field. In recent years, we have focused on the development of an osteopathic care pathway. In this paper, we provide a detailed description of this procedure, the development of the method and its clinical application and validity. The ultimate goal is to improve effectiveness in the use of osteopathy in neonatology practice and make the use of a standardized approach more efficient.

## Methods

### The development of the NE-O model

An Osteopathic model in Neonatology ward (NE-O model) was designed from the NE-O group of the Accademia Italiana Osteopatia Tradizionale (AIOT). The aim of the NE-O model was to codify an osteopathic procedure to increase reliability, internal and external validity, efficiency and accuracy of osteopathic diagnosis and treatment on newborns. The NE-O model was tested on newborns, both preterm and term infants, of either sex with parents’ or legal guardians’ written informed consent. No exclusion criteria were applied. It is composed of a set of evaluation and treatment procedures. The duration of an osteopathic session lasts 30 minutes, 10 min for evaluation and 20 min for treatment. The model has been developed following two phases: a run-in pre-test period and a research period. The 8-month run-in period was carried out between April 2006 and December 2006. It consisted of pre-test evaluation and treatment of 100 newborns by 3 licensed osteopaths (mean ± SD: 43.6 ± 0.57 years, 1 man and 2 women), with a mean of 9.6 ± 4.0 years of clinical experience and same osteopathic curricula. Osteopaths were following the same evaluation procedure and treatments. The techniques of choice were balanced ligamentous and membranous tension techniques. After the run-in period, the model was applied to research context and results from different clinical trials were published elsewhere [7-9].

### Evaluation procedures

A specific osteopathic evaluation has been designed and described as follows. The first step in newborns’ evaluation is to look at the general condition of the child in terms of asymmetries and defects of posture.

Then, the operator takes place beside or aside the crib of the newborn. The assessment starts from the skull, continuing with the spine and the pelvis passing through upper and lower limbs and ends with the rib cage and viscera. The evaluation is performed according to TART (Tissue alteration, Asymmetry, Range of motion and Tenderness) criteria [10] aiming to locate SD. During the evaluation process, osteopaths applied only passive tests as they do not require the active collaboration of the subject.

#### Skull

The skull is assessed in order to detect any cranial strain pattern (CSP), condylar compression and suture/fontanelles abnormalities. The position of the operators is aside to the child.

**Figure 1:**
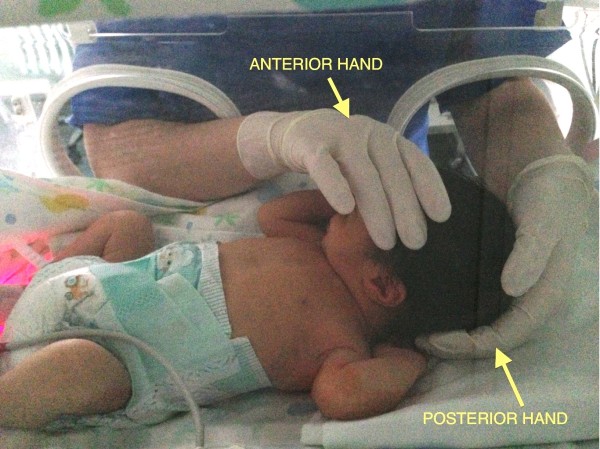
**Modified frontal-occipital hold.** The osteopath performs a frontal-occipital hold placing the anterior hand on the frontal bone and the posterior hand under the occiput. The operator stands aside the crib.

**Figure 2:**
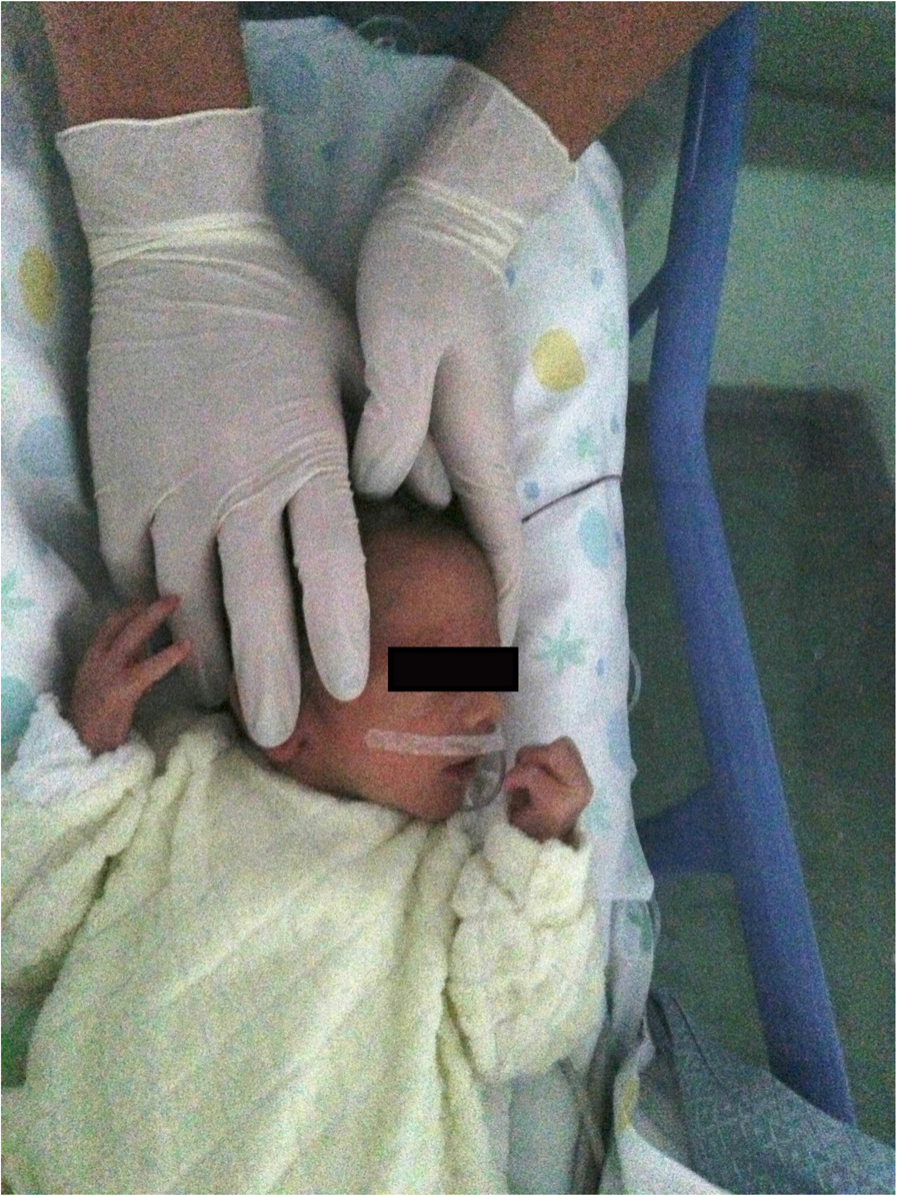
**Modified five fingers hold.** The osteopath performs a modified five fingers hold using three or four fingers according to newborns’ skull size. The operator is positioned at the head of the infant.

Firstly a general CSP assessment is performed with a modified fronto-occipital hold and a modified five fingers hold. The former is performed putting the first and the second finger of one hand (anterior hand) upon the greater wings of the sphenoid bone and the other hand (posterior hand) on the occiput (occiput hold) (Figure 1). The modified five fingers hold is performed using only three or four fingers of both hands (according to skull size) as following: indexes are placed on the greater wings of sphenoid bone, thumbs are put on the vault and middle fingers are located on the asterion (Figure 2). In these ways is possible to assess the primary respiratory mechanism (PRM) and diagnose the presence or absence of spheno-basilar syncondrosis (SBS) compression and/or any other dysfunctional CSP.

**Figure 3:**
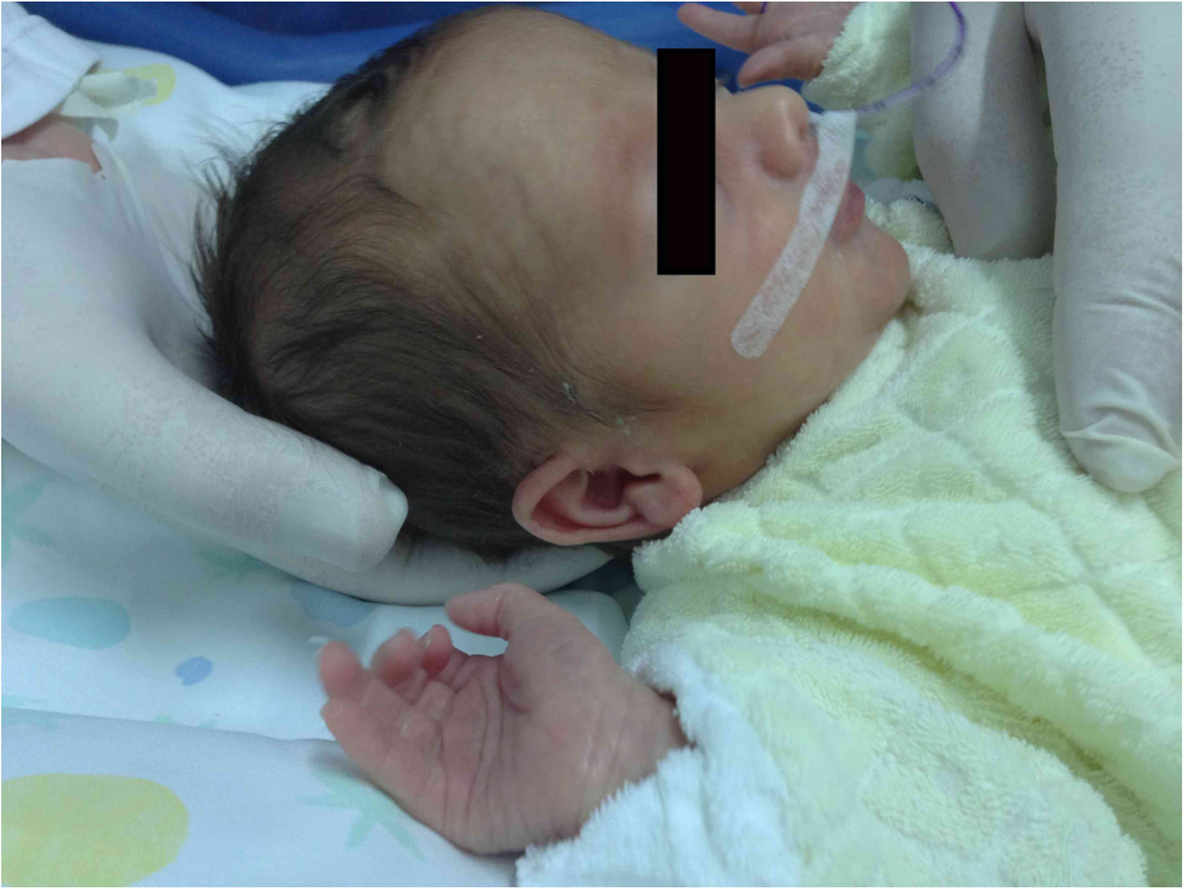
**Occiput condyles hold.** The osteopath carries out an occiput condyles hold, monitoring with two fingers the condyles restriction.

Secondly occipital condylar evaluation is carried out using the second and the fourth fingers of only one hand unlike the traditional hold performed with both hands (Figure 3).

Thirdly, a sutures and fontanelles evaluation is conducted to state if there are overlapping margins or premature fusing of cranial sutures. Due to fragile newborns conditions the suture evaluation is restricted to external sutures only.

#### Spine

**Figure 4:**
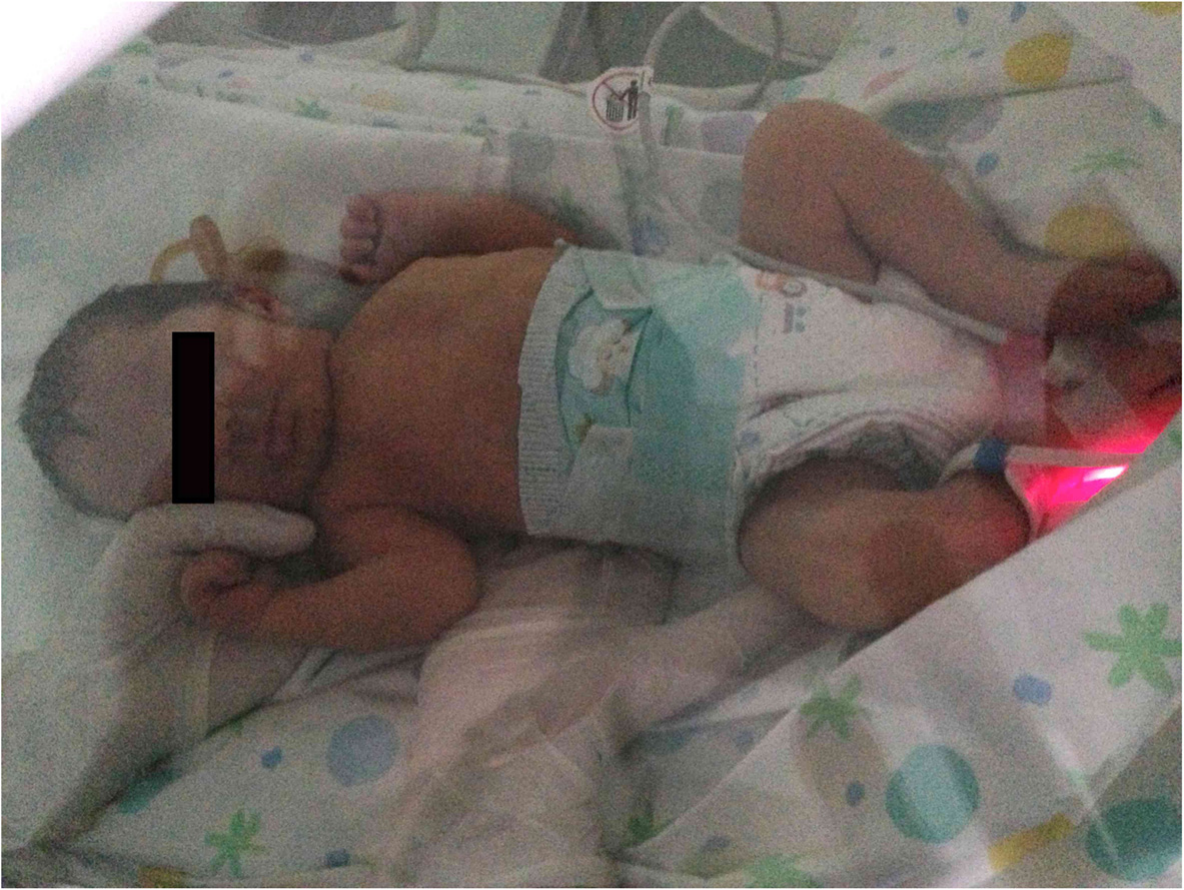
**Modified spring test for the column.** The osteopath performs the modified spring test standing asides to the newborn and placing both hands under the back of the subject. A light pressure is applied over the spinous processes to investigate the presence of SD.

The test of choice for the assessment of the spine is a modified spring test. The traditional spring test is performed with patient in prone position in order to investigate the presence of an anterior torsion or a unilateral flexion of the sacrum [11]. In neonates is performed lying on the table in supine position on the entire spine. The operator, aside to the child, places both hands under the back of the baby and with fingertips applies light pressure over the spinous processes to investigate the presence of SD (Figure 4).

#### Pelvis

**Figure 5:**
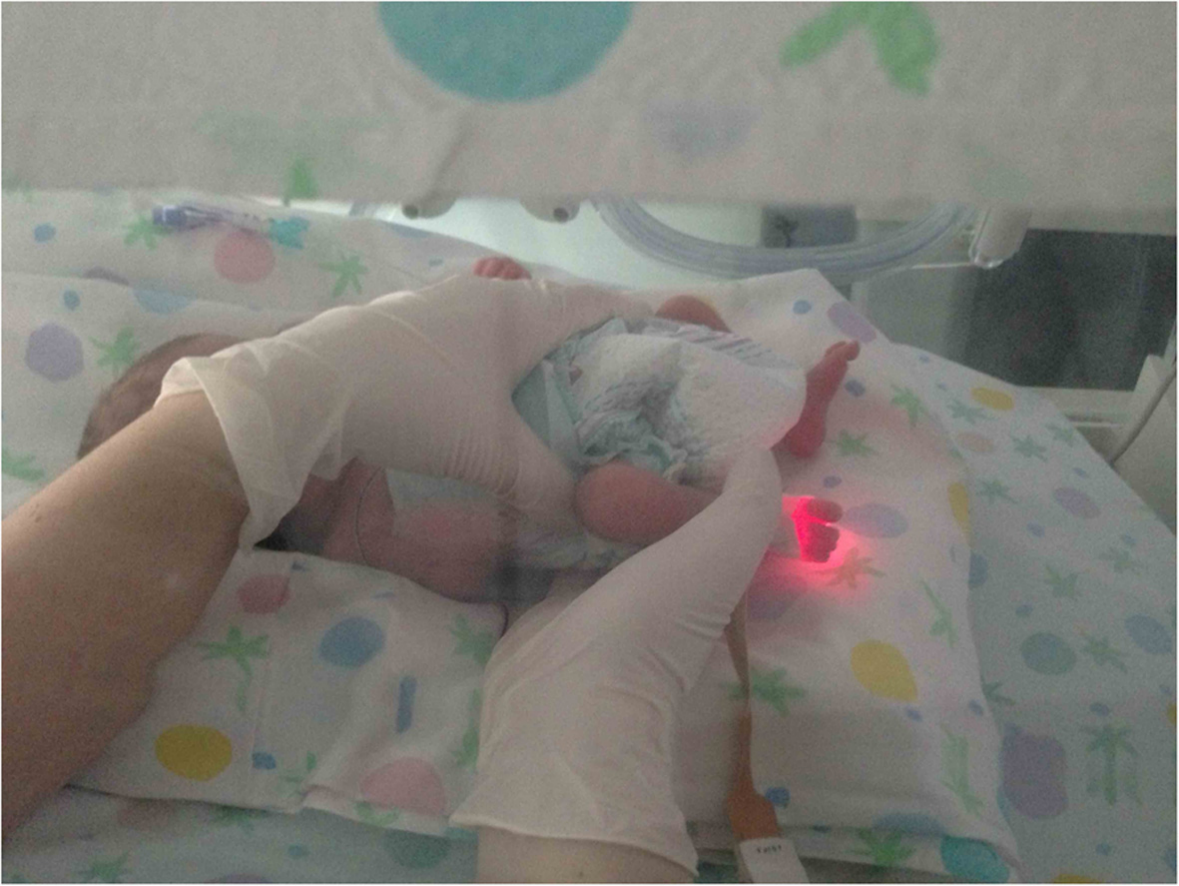
**Sacroiliac hold.** The osteopath is positioned aside from the crib and performs a sacroiliac hold in order to diagnose any compression or restriction of mobility.

The operator always stands next to the child and with a sacroiliac hold performs the pelvis assessment in order to evaluate the presence of any sacrum intraosseous lesion, any sacrolumbar or sacroiliac compression and pubic dysfunction (Figure 5).

#### Upper limbs

The assessment of the upper limbs has the aim to investigate the articular range of motion in each joint. Examinations are passive and specific for each joint, illustrated as follows:

Shoulder: small and delicate movements is required to evaluate flexion-extention, ab-adduction, internal and external rotation;

Elbows: internal and external rotation and flexion-extention test is performed;

Wrists: flexion-extention test is carried out.

#### Lower limbs

As for the upper arms, the assessment of the lower limbs consists of a passive test performed to evaluate the range of motion. They can be described according to areas as follows:

Hips: the range of motion in the three axes planes is tested: flexion-extention, ab-adduction, internal and external rotation;

Knee: flexion-extention test of knee joint and internal-external rotation of the tibia are performed;

Ankle: flexion-estention test of tibio-tarsal joint is conducted.

#### Rib cage

**Figure 6:**
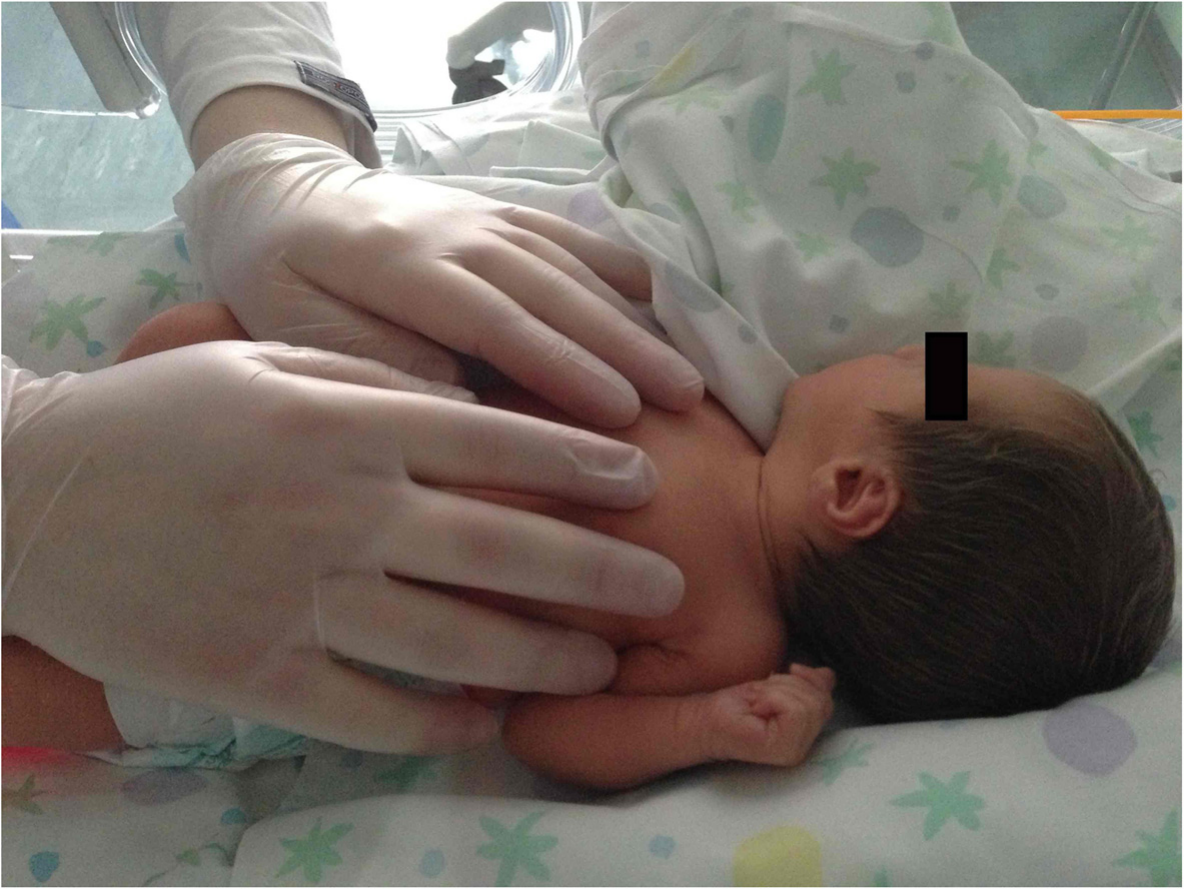
**Rib cage assessment.** The osteopath carries out a rib cage assessment placing both hands on the rib cage and applying a gently pressure in order to evaluate resistance, elasticity and mobility of the ribs.

The operator places the hands over the chest of the newborns. A gentle pressure on the rib cage is performed in order to evaluate resistance, elasticity and mobility of the ribs (Figure 6).

#### Diaphragm

The operator stands aside the newborn and places the anterior hand on the diaphragmatic cupola and the posterior one on the diaphragmatic pillars testing the area for restrictive barrier and tissue alteration.

#### Viscera

**Figure 7:**
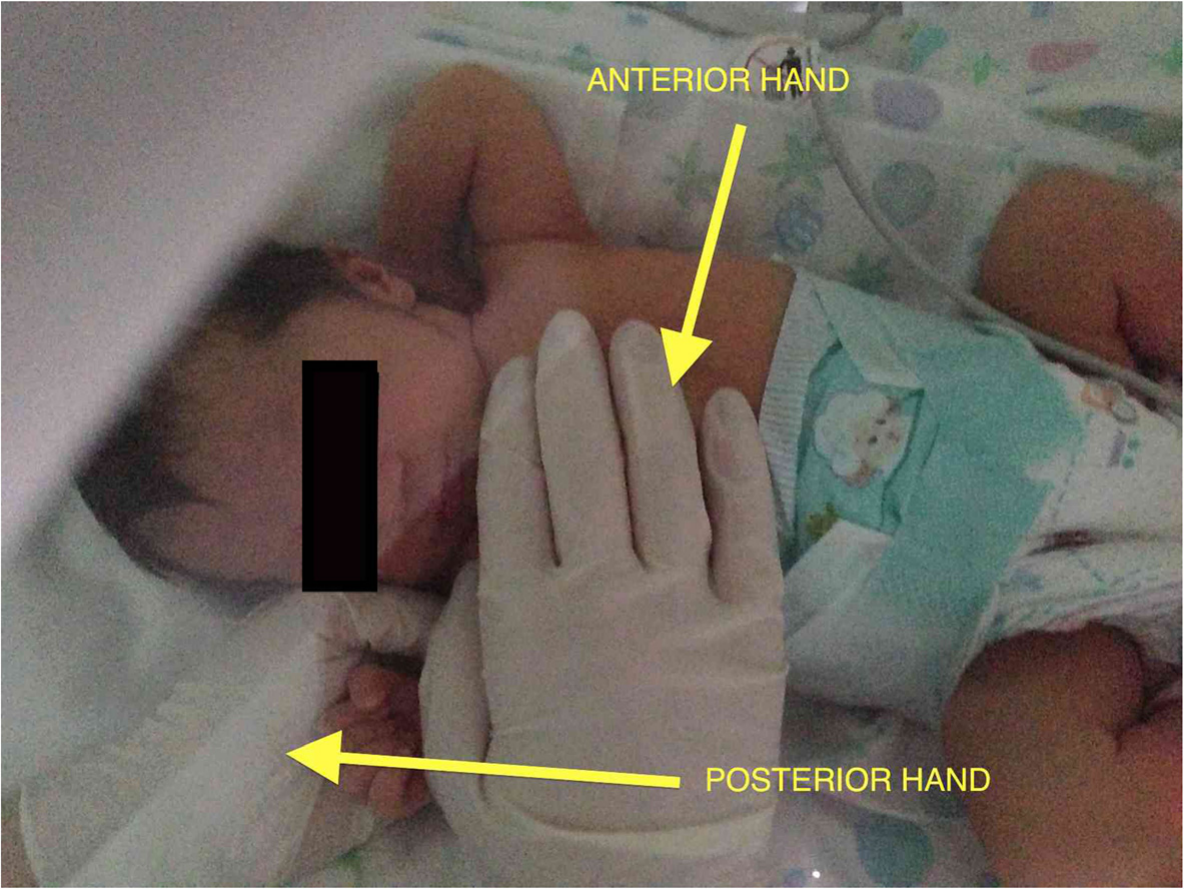
**Visceral fascia assessment.** The osteopath assesses the visceral fascia considering the relation between the range of motion of tissues of the anterior area and the referred dermatomeric area of the column. Anterior hand is placed on the visceral region whilst the posterior one is located on the column.

The assessment ends with the evaluation of the visceral fascia. It was decided to approach the visceral fascia by dividing it into three different regions: the anterior region of the neck, the mediastinal region and the abdominal-pelvic region. The assessment considers the relation between the range of motion of tissues of the anterior area and the referred dermatomeric area of the column. One hand (anterior) is placed on the visceral region whilst the other hand (posterior) is located on the column (Figure 7). The anterior tissue is tested in every direction (caudally, cranially, to the left and to the right) with gentle maneuvers, looking for the restrictive barrier; contextually the other hand monitors the motility of the column corresponding to the sympathetic innervation. Specifically, this approach is described according to area as follows:

Anterior region of the neck:

The operator places one hand (anterior hand) over the throat and the other hand (posterior hand) under the cervical spine looking for movement restriction and tissue alteration.

Mediastinum:

**Figure 8:**
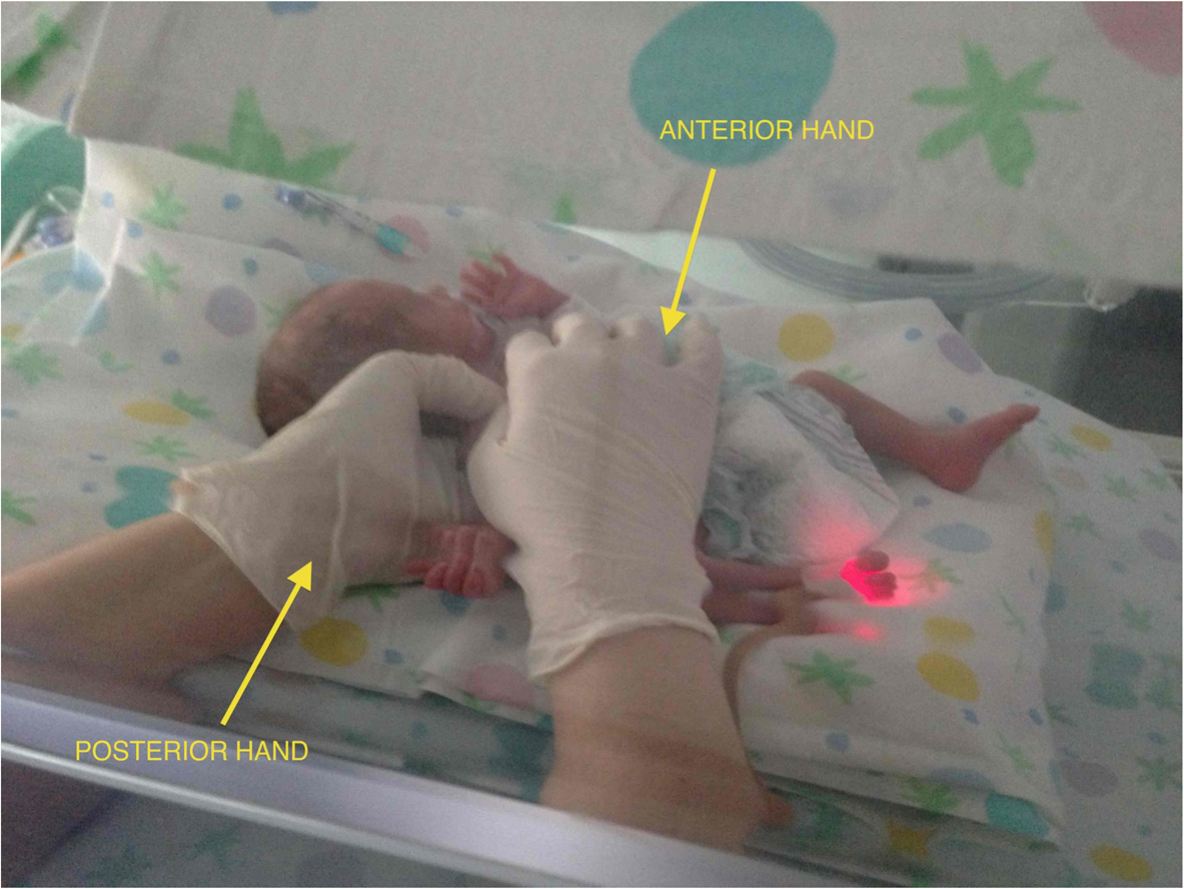
**Mediastinum assessment.** The osteopath evaluates the mediastinum placing the anterior hand on the sternum and the posterior one under the dorsal spine, between T1-T6.

The operator puts one hand (anterior hand) over the sternum and the other hand (posterior hand) on the dorsal spine covering the area between T1 - T6 (Figure 8).

Abdominal-pelvic region:

**Figure 9:**
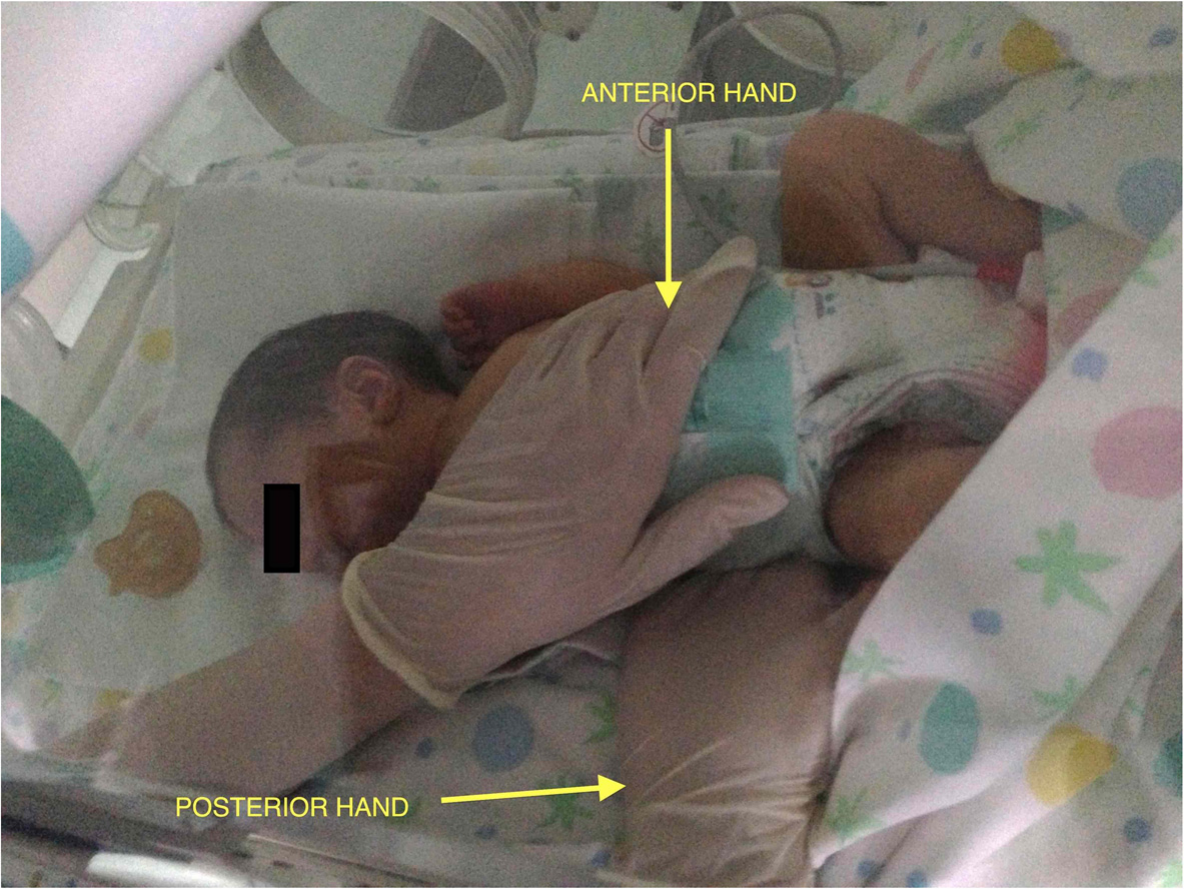
**Abdominal-pelvic assessment.** The osteopath performs the abdominal-pelvic assessment placing the anterior hand on the abdomen and the posterior one on the thoracolumbar tract.

The operator puts the anterior hand over the subject’s belly and the posterior hand on the thoracolumbar tract (Figure 9).

### Treatment procedures

**Figure 10:**
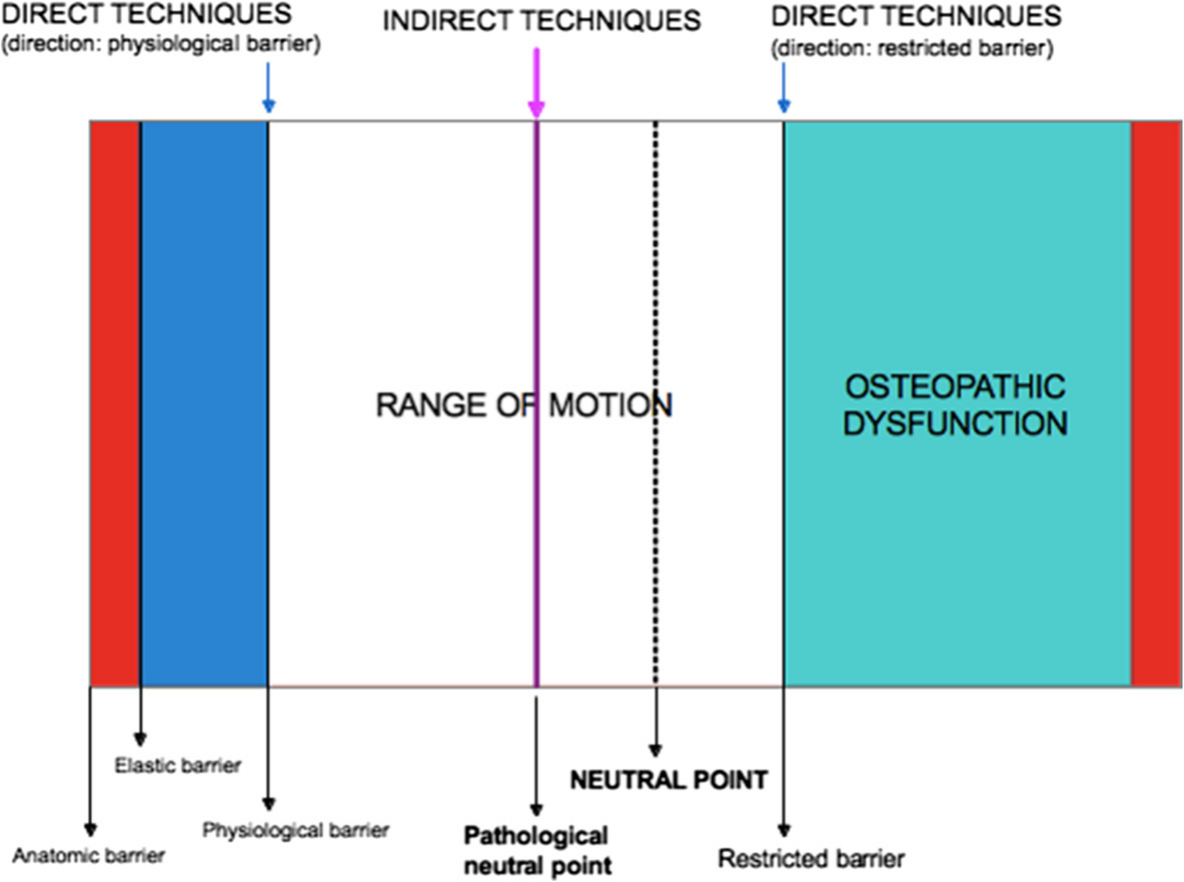
**Barriers, range of motion and neutral points in somatic dysfunctional areas.** Direct techniques are applied on either the physiological (i.e. myofascial release technique) or restricted barrier (high- velocity low-amplitude, muscle energy, articulatory and soft tissue techniques). Indirect techniques (functional, BLT and BMT) are focused on the pathological neutral point.

The second phase of the NE-O model is based on treatment. The term osteopathic manipulative treatment (OMT) currently includes nearly twenty-five types of manual treatments. These techniques are used to treat SD within the body’s framework, including skeletal, arthrodial, and myofascial structures and associated vascular, lymphatic and neural components [1]. The OMT techniques of choice in treating preterm infants are indirect techniques (counterstrain, cranial, facilitated positional release, functional, balanced ligamentous tension) [1,10] (Figure 10). Those are manipulative techniques where the restrictive barrier is disengaged; the dysfunctional body part is moved away from the restrictive barrier until tissue tension is equal in all planes and directions.

In Sutherland’s original thinking, the ability of healing is handled by intrinsic forces of the body, rather then to the action of the operator [12]. The indirect approach exploits these forces and potentially is more effective on newborns because it works on the still point and on the intrinsic movement of the structures. Additionally, it does not require the cooperation of the patient [1].

Specifically the treatment method used follows Sutherland’s point of maximum freedom (balance point) model. According to Sutherland’s model, all the joints in the body are balanced ligamentous articular mechanisms. The attention is focused on the balance point defined as the point in the range of motion of a joint where the anatomical structures (ligaments, membranes and other mesenchymal structures) laid in maximum freedom and minimal tension. This is usually between the normal tension present in a rest position and the increased tension preceding the strain or fixation which occurs when a joint is carried beyond its normal physiology. Therefore it is the most “neutral” position possible under the influence of all factors responsible for strains. SD tends to alter the balance of ligamentous framework. Consequently the first step of an indirect treatment procedures is to determine the point of balance. To do this it is necessary to gently test the area with SD, in every direction within the existing range of motion, seeking for tissue barriers and eventually reaching the point of balance [13].

Balanced Ligamentous Tension (BLT) and Balanced Membranous Tension (BMT) techniques follow these principles and thus are the techniques of choice in the present osteopathic model (Table 1). These techniques have several additional advantages. Firstly the active participation and the mobilization of the patient is not requested. Secondly, supine posture is the preferred position to perform the techniques. Finally, infants’ safety can be warranted as BLT and BMT are potentially less aggressive than other techniques. As lack of evidence, it should be pointed that the latter two points derived from clinical considerations and experience, thus not supported by rigorous scientific evidence. Moreover, it should be highlighted that some traditional BLT and BMT techniques should be modified according to the needs of the babies.

**Table 1:** Steps of BLT and BMT techniques according to Sutherland’s description

**Phase**	**Operator status**	**Description**
1. Disengagement	ACTIVE	The practitioner uses a compression or decompression method to disengage the area with SD.
2. Reaching the balance point	ACTIVE	The area or the segment with SD is moved through the existing range of motion in every direction, with close attention to whatever restriction may be present.
3. Balance point	PASSIVE	The dysfunctional area is brought in the balance point and the body’s inherent power is monitored.
4. Tissue release	PASSIVE	An increase of local temperature and a rebalance of joint mobility is achieved.

### Techniques description

#### Balanced Ligamentous Tension (BLT)

According to Sutherland’s theory, ligaments provide both proprioceptive information that guides muscle response for joint positioning and the anatomical framework to control motion of the articular components [12]. BLT approach starts looking for the balance point of the joint or the part of the body where SD has been diagnosed. The assumption is that putting the area with SD under the lowest possible strain, the body’s inherent power can operate effectively on the tissue to restore functional freedom. A point of “neurological neutrality” is achieved, and probably the central nervous system reacts producing an undiscovered cascade of events that create the biological conditions for SD solving (Table 1). Each indirect technique that use this mechanism can be regarded as based on the principle of “afferent reduction” [13].

#### Balanced Membranous Tension (BMT)

BLT techniques applied to CSP are referred as BMT. The anatomical component of BMT are the cranial bones and indirectly the dura mater; more precisely the relationship between flax cerebri, tentorium and flax cerebelli described by Sutherland as reciprocal tension membranes [13]. Although scientific evidence is lacking, osteopathic clinical experience and tradition consider the cranial function a keynote mechanism. As for BLT, the first step of BMT techniques is to contact the cranial bones with SD. Afterword the point of balance is sought, maintained and the correction obtained [13] (Table 1).

### Data collection

Contextually to the development of the NE-O model, an ad-hoc software (EBOM-GCCN) has been built to improve efficiency and accuracy of data as well as to support osteopaths and NICU stuff in patients’ management. Specifically, two of the three sections were designed to collect osteopathic evaluation and treatment data, respectively.

### Ethics statement

Written informed consent was obtained for each subjects and the use of the model was approved by the institutional review board of Pescara’s hospital. The model has been registered at http://www.clinicaltrials.gov (identifier number: NCT01902563).

## Results

The NE-O model was developed in April 2006 to evaluate the efficacy of OMT in neonatology. Infants enrolled included preterm and term newborns admitted to the NICU of the civil hospital of Pescara, Italy.

During the entire study period (2006 - present), the model has been tested on over 2000 hospitalized preterm and term newborns both healthy and clinically complicated as well as on neonates with congenital and genetic conditions. No adverse events have been recorded, suggesting that the model is safe.

The effectiveness of this model was demonstrated elsewhere [7-9] and it is not the intent of this paper to discuss it.

## Discussion

The present paper has the aim to introduce an osteopathic evaluation and treatment model within the NICU. Results from recent studies documented the effectiveness and safety of the model.

Nevertheless, here we can describe our first experiences with the NE-O model and make recommendations for future practice. Since 2006 we have undertaken comprehensive research with the aim of creating an osteopathic standardized procedure that could systematically improve the use of osteopathy as adjutant therapy in neonatology. The NE-O procedures appears to be an efficient method to achieve this goal. In the context of using osteopathic approach in clinical practice, it is noteworthy to consider some issues. Infants anatomy is different from that on adults. This difference should be taken into account especially for the cranial approach. In newborns, sutures are not fully developed as well as bones, as justified by the presence of fontanelles. For this reason, traditional cranial treatment on sutures should be adapted to infants’ skull. A global approach to the cranium (fronto-occipital, modified five fingers or cranial fossae hold) could be considered the preferred way to assure safety and increase effectiveness of the treatment. Direct sutural techniques may be biased from incomplete skull development.

In addition, the visceral model proposed has been built upon clinical and anatomical consideration. Looking at the osteopathic literature, the only visceral approaches described are those providing a direct contact with the organ (direct approach) [14], however, no published data is available to confirm the safety and applicability in the field of neonatology. In the context of NICU, the choice of a different visceral approach was made on three essential reasons. Firstly, considering the medical semiotics there is no examination that allows a direct and specific palpation of visceral structures. Only few parenchymal organs are approachable in relation to the abdominal wall. Moreover, the ability to palpate a visceral structure is symptomatic of a suspected pathological condition [15]. Secondly, a direct approach on newborn could be dangerous because of their fragile conditions. Finally, the small size of infant anatomy makes osteopathic practitioners unable to perform traditional approaches.

Another peculiarity about the NE-O model is the osteopathic evaluation. It is tailored to hospitalized infants and differs from that on adults mainly for patient’s position (only the laying down posture is allowed) and type of tests used (only passive tests are applicable). Furthermore, the model fits the needs of all NICU levels.

Therefore the NE-O model has the strengths to be clinically validated, effective, safe, methodologically reproducible and applicable into all NICU levels. However, due to lack of standardized osteopathic procedures in neonatology, the NE-O model could not be compared to pre-existing methods.

Interestingly, the application of this model into a neonatology unit could be considered one of the first real osteopathic example of complex intervention. The latter is widely used in health services, public health practices and health-related policies and it is mainly based on synergic and integrated collaborations. To optimize the osteopathic intervention within an integrated care system, osteopathic session should be included into a precise medical care timetable, without interfering with routine medical and paramedical procedures. Examples came from the NE-O study where osteopathic care is administered early afternoon before the visiting hours and after the medical and paramedical daily assistance [7,8]. The collaboration between NICU staff and osteopaths to deliver more accurate health care services is another key point. Making osteopaths informed about childhood diseases, allows them to perform more precise evaluations and treatments towards the somatic areas responsible for clinical symptoms. Contextually, keeping medical staff informed about dysfunctional areas can broaden medical semeiotics optimizing newborns medical cares. Unpublished data from the NE-O study documented this successful collaboration (Cerritelli F, Barlafante G, Renzetti C, Pizzolorusso G, Cozzolino V: Collaborative care into neonatalogy ward: and osteopathic example, in preparation). Therefore, the inclusion of osteopathic care in neonatology ward can be considered a good example of integrated medicine and complex intervention.

## Conclusion

This paper provides a method for the osteopathic care of infants admitted to neonatal intensive care unit. Specifically, it aims to provide an osteopathic action plan for evaluation and treatment in the specific field of neonatology. Moreover it proposes an osteopathic model in terms of newborns’ evaluation and treatment.

The success of the methodology proposed is supported by some positive experiences with the use of the NE-O model in different NICUs across Italy. Results from these research showed the effectiveness of this osteopathic model in reducing preterms’ length of stay and hospital costs. Additionally the present model has been demonstrated to be safe.

Therefore the standardization of the procedure could be a keynote step to optimize the osteopathic approach and its potential benefit in neonatal field as well as to make the method reproducible in NICU setting.

## Abbreviations

BLT: Balanced ligamentous tension; BMT: Balanced membranous tension; CAM: Complementary and alternative medicine; CSP: Cranial strain pattern; NICU: Neonatology intensive care unit; OMT: Osteopathic manipulative treatment; SD: Somatic dysfunction; TART: Tissue alteration, asymmetry, range of motion and tenderness.

## Competing interests

The authors declare that they have no competing interests.

## Authors’ contributions

FC and MM wrote the paper. GB, VC, CR and GP reviewed the paper for important intellectual content. All authors read and approved the final manuscript.

## References

[B1] American Association of Colleges of Osteopathic Medicine (AACOM) Glossary of Osteopathic Terminology 2011 Chevy Chase, MD: AACOM

[B2] Lund GC Edwards G Medlin B Keller D Beck B Carreiro JE Osteopathic manipulative treatment for the treatment of hospitalized premature infants with nipple feeding dysfunction J Am Osteopath Assoc 2011 111 44 48 21258016 10.7556/jaoa.2011.111.1.44

[B3] Wescott N The use of cranial osteopathy in the treatment of infants with breast feeding problems or sucking dysfunction Aust J Holist Nurs 2004 11 25 32 19175274

[B4] Andreoli ET Troiani A Tucci B Barlafante G Cerritelli F Pizzolorusso G Renzetti C Vanni D Pantalone A Salini V Osteopathic manipulative treatment of congenital talipes equinovarus: a case report J Bodyw Mov Ther 2014 18 1 4 10 10.1016/j.jbmt.2013.03.011 24411143

[B5] Friedman SJ Osteopathic manipulation: promise for infantile colic J Am Osteopath Assoc 2008 108 483 18806075

[B6] Lessard S Gagnon I Trottier N Exploring the impact of osteopathic treatment on cranial asymmetries associated with nonsynostotic plagiocephaly in infants Complement Ther Clin Pract 2011 17 193 198 10.1016/j.ctcp.2011.02.001 21982132

[B7] Pizzolorusso G Turi P Barlafante G Cerritelli F Renzetti C Cozzolino V D’Orazio M Fusilli P Carinci F D'Incecco C Effect of osteopathic manipulative treatment on gastrointestinal function and length of stay of preterm infants: an exploratory study Chiropr Man Therap 2011 19 15 10.1186/2045-709X-19-15 21711535 PMC3155103

[B8] Cerritelli F Pizzolorusso G Ciardelli F La Mola E Cozzolino V Renzetti C D’Incecco C Fusilli P Sabatino G Barlafante G Effect of osteopathic manipulative treatment on length of stay in a population of preterm infants: a randomized controlled trial BMC Pediatr 2013 13 65 10.1186/1471-2431-13-65 23622070 PMC3648440

[B9] Cerritelli F Pizzolorusso G Renzetti C D’Incecco C Fusilli P Perri PF Tubaldi L Barlafante G Effectiveness of osteopathic manipulative treatment in neonatal intensive care units: protocol for a multicentre randomised clinical trial BMJ open 2013 3 e002187 23430598 10.1136/bmjopen-2012-002187 PMC3586056

[B10] Ward RC Foundations for Osteopathic Medicine 2002 2 Baltimore, MD: Lippincott Williams & Wilkins

[B11] De Stefano LA Greenman’s Principles of Manual Medicine 2011 Baltimore, MD: Lippincott Williams & Wilkins

[B12] Sutherland WG Wales AL Teachings in the Science of Osteopathy 1990 Portland, OR: Rudra press

[B13] Magoun HI Osteopathy in the Cranial Field 1976 3 Kirksville, MO: Journal Priming Co

[B14] Barral J-P Mercier P Visceral Manipulation 2005 Seattle: Eastland Press

[B15] AAVV Manuale di segni e sintomi 2010 Roma: PICCIN

